# Asian flush is a potential protective factor against COVID-19: a web-based retrospective survey in Japan

**DOI:** 10.1265/ehpm.23-00361

**Published:** 2024-03-09

**Authors:** Satoshi Takashima, Mikiko Tokiya, Katsura Izui, Hiroshi Miyamoto, Akiko Matsumoto

**Affiliations:** 1 Department of Social and Environmental Medicine, Saga University, 5-1-1 Nabeshima, Saga 849-8501, Japan; 2 Plant Products Safety Division, Food Safety and Consumer Affairs Bureau, Ministry of Agriculture, Forestry and Fisheries, 1-2-1 Kasumigaseki, Chiyodaku, Tokyo 100-8950, Japan; 3 Graduate School of Biostudies, Kyoto University, Oiwake-cho, Kitashirakawa, Sakyo-ku, Kyoto 606-8502, Japan; 4 Department of Pathology and Microbiology, Faculty of Medicine, Saga University, 5-1-1 Nabeshima, Saga 849-8501, Japan

**Keywords:** Asian flush, COVID-19, ALDH2, rs671

## Abstract

**Background:**

Coronavirus disease 2019 (COVID-19), first reported in December 2019, spread worldwide in a short period, resulting in numerous cases and associated deaths; however, the toll was relatively low in East Asia. A genetic polymorphism unique to East Asians, Aldehyde dehydrogenase 2 rs671, has been reported to confer protection against infections.

**Method:**

We retrospectively investigated the association between the surrogate marker of the rs671 variant, the skin flushing phenomenon after alcohol consumption, and the timing of COVID-19 incidence using a web-based survey tool to test any protective effects of rs671 against COVID-19.

**Results:**

A total of 807 valid responses were received from 362 non-flushers and 445 flushers. During the 42 months, from 12/1/2019 to 5/31/2023, 40.6% of non-flushers and 35.7% of flushers experienced COVID-19. Flushers tended to have a later onset (Spearman’s partial rank correlation test, p = 0.057, adjusted for sex and age). Similarly, 2.5% of non-flushers and 0.5% of flushers were hospitalized because of COVID-19. Survival analysis estimated lower risks of COVID-19 and associated hospitalization among flushers (p = 0.03 and <0.01, respectively; generalized Wilcoxon test). With the Cox proportional hazards model covering 21 months till 8/31/2021, when approximately half of the Japanese population had received two doses of COVID-19 vaccine, the hazard ratio (95% confidence interval) of COVID-19 incidence was estimated to be 0.21 (0.10–0.46) for flusher versus non-flusher, with adjustment for sex, age, steroid use, and area of residence.

**Conclusions:**

Our study suggests an association between the flushing phenomenon after drinking and a decreased risk of COVID-19 morbidity and hospitalization, suggesting that the rs671 variant is a protective factor. This study provides valuable information for infection control and helps understand the unique constitutional diversity of East Asians.

**Supplementary information:**

The online version contains supplementary material available at https://doi.org/10.1265/ehpm.23-00361.

## 1. Background

Coronavirus disease 2019 (COVID-19) caused by SARS-CoV-2 was first reported in Wuhan, China, in 2019. The infection spread to other countries within a short period and became a global pandemic. Until 2021, the COVID-19 incidence and mortality rate in the Western Pacific region, including Japan, were lower than those in other areas, such as Europe and the US [1, 2]. As of August 31, 2021, the Czech Republic ranked the first in terms of the cumulative incidence of COVID-19 per 100,000 people among the 38 OECD member countries, with 16,170 cases, whereas Japan ranked thirty-fifth with 1,193 cases (Fig. S1). In terms of cumulative COVID-19-related deaths, Hungary ranked first with 310 deaths/100,000, and Japan ranked thirty-fourth with 12.9 deaths/100,000 (Fig. S1). Since 2022, the incidence rate in Japan has increased and is currently higher than that in Hungary. However, the mortality rate in East Asia, including Japan, has been reported to be low (Fig. S1). Possible factors associated with the low incidence and mortality rate of COVID-19 in Japan, especially in the initial stages of the pandemic, have been discussed, including strict usage of masks, a cultural background of low interpersonal contact, and high hygiene awareness; however, genetic factors may also be involved.

East Asia has a high prevalence of the single-nucleotide polymorphism of the aldehyde dehydrogenase 2 (*ALDH2*) gene, rs671 [3, 4], whose variant alleles produce region-specific disease structures [5–7]. The rs671-caused amino acid substitution of Glu504Lys (or Glu487Lys in the mature protein) reduces enzyme activity, mainly by disrupting the dimer structure. The mutant and wild-type monomers randomly form tetramers, resulting in a less intact ALDH2 protein, with 16% of the estimated enzyme activity for heterozygotes and 0% for homozygotes of the variant allele [5, 7]. As ALDH2 is the only enzyme that can metabolize acetaldehyde at low concentrations, most rs671 variant carriers experience uncomfortable symptoms such as skin flushing, the so-called “Asian flush,” due to elevated blood levels of acetaldehyde after consuming alcoholic beverages [8]. The variant allele reduces the prevalence of habitual drinking and alcohol-related diseases; however, habitual drinking by variant carriers is associated with a higher risk of esophageal cancer than in non-carriers [9]. Thus, ALDH2 is recognized as a drinking-related enzyme; however, the essential role of ALDH2 is the metabolism of other types of endogenous aldehydes as ALDH2 is expressed in various species, including algae, fish, and rodents [10]. For example, formaldehyde and 4-hydroxy 2-nonenal (4-HNE) are well-known endogenous aldehydes and substrates of ALDH2 [11–13]. Formaldehyde is an essential aldehyde produced via different pathways, such as one-carbon metabolism [14] and amino acid metabolism [15], and plays a role in promoting macrophage function [16, 17] and inhibiting bacterial growth [18]. 4-HNE is a highly toxic and abundant stable end-product of lipid peroxidation that is implicated in aging and other pathological states [19, 20], and is also an effective bacteriostatic agent [18]. These findings suggest a reason for the evolutionary expansion of rs671 [21–23], and the reduced activity of ALDH2 is reportedly protective against bacterial infections, in both epidemiological studies and animal models [18, 24]. The bacteria reported are intracellular microorganisms such as *Mycobacterium tuberculosis*, which may activate common immunological pathways with SARS-CoV-2, such as Toll-like receptor 9, which is expressed on endosomes and the cell surface of various immune cells, including B cells and macrophages [25–27].

Here, we designed a web-based retrospective survey to test the hypothesis that the rs671 variant protects against COVID-19. As the occurrence of “Asian flush” around the time of the first drink indicates the presence of variant allele of rs671 with an accuracy of approximately 90% [28–30], we used this skin flushing phenomenon as a surrogate marker for the variant allele of rs671.

## 2. Methods

### 2.1. Participants

The survey was conducted from August 7 to 27, 2023, using Google Forms (Googleplex, Mountain View, CA, USA) (www.google.com/intl/ja_jp/forms/about/), which were used to create a web-based survey site. Social media platforms, such as Facebook and LINE, were used to encourage voluntary participation. Prior to data collection, details of the survey methodology, including background, purpose, outline, and ethical considerations, were disclosed, and consent of the respondents was acquired (the only option to proceed with the survey). Among the 811 respondents, 807 (367 female and 440 male individuals) were included in the analysis, after excluding 1 person living outside Japan, 1 under 20 years of age, and 2 who could not remember the time of infection. The response rate was unknown because the URL to the survey site was posted on a social media platform without restrictions on viewers. The project was conducted as planned after review and approval by the Ethics Committee of the Saga University School of Medicine, which was established in accordance with the Declaration of Helsinki and Japanese Government guidelines and includes experts in humanities, ethics, and law (Reception No. R5-11).

### 2.2. Questionnaires

All questions listed in Table S1 were asked only in Japanese. Briefly, the main questions were about the skin flushes around the time of the first drink, number of COVID-19 experiences, time of disease development (by selecting the appropriate time frame in 3-month intervals), basis for diagnosis (evidence for COVID-19), and experience with hospitalization due to COVID-19. Additional questions included biological sex, ethnicity (Japanese, other East Asian, or other), prefecture of residence, occupation, age group (in 10-year increments), body mass index (BMI), smoking habit, drinking habit, amount of alcohol consumption, medical history, steroid medication status, and number of COVID-19 vaccine doses. Bodyweight was not included in the questionnaires to increase participation because some participants in the preliminary survey refused to provide their weight. Hokkaido, Tokyo, Kanagawa, Aichi, Osaka, Kyoto, and Fukuoka were defined as endemic residential areas, whereas the other 40 prefectures were defined as non-endemic areas. Habitual drinkers were defined as those who consumed alcohol more than once a week. Average daily ethanol consumption was calculated and adjusted for body weight of 60 kg and multiplied by a factor of 60/63 for male individuals and 60/51 for female individuals, based on the average body weight of the Japanese population (male, 63 kg; female, 51 kg), and then divided into three groups: 0, ≤20, and >20 g per day.

### 2.3. Statistical analysis

Chi-square test or Fisher’s exact test was used to detect differences in distribution between non-flushers and flushers.

#### 2.3.1. Outcomes and explanatory variable

Primary and secondary outcomes were the first incident of COVID-19 and experience of hospitalization due to COVID-19, respectively. The explanatory variable was flushing, defined by self-reported skin flushing around the time of the first drink.

#### 2.3.2. Survival curves

Survival time analysis was performed for the entire 42-month observation period (12/1/2019–5/31/2023), stratified using the flushing characteristic (non-flusher or flusher), with the event being the first incident of COVID-19 or the experience of hospitalization due to COVID-19 (Generalized Wilcoxon test with PROC LIFETEST; SAS9.4 for Windows, SAS Institute, Cary, NC, USA).

#### 2.3.3. Hazard ratio

We attempted to estimate multivariate-adjusted hazard ratios for COVID-19 incidence using the Cox proportional hazards model (PROC PHREG, SAS9.4) and found that proportional hazards did not hold over the entire 42-month observation period (Fig. S2), raising the possibility of a strong influence of vaccination or group immunity on the events. As we could not control for these effects, the first 21 months (12/1/2019–8/31/2021; the period during which many Japanese residents had not completed two doses of the vaccine (Fig. S3)) were used as the observation period. The primary explanatory variables were flushing characteristics and the covariates were sex, age group, steroid use, area of residence (endemic or non-endemic), occupation, and drinking habits.

#### 2.3.4. Sensitivity analysis

To check the robustness of the Cox proportional hazards model, sensitivity analyses were performed by considering cases reported as self-diagnosed as unaffected and additional covariates of BMI, sleep disorders, medical history (diabetes, heart disease, allergic disease, collagen disease, respiratory disease, and hepatic disease), and smoking habit. The interactive term of drinking habit and flushing, followed by a single term of drinking, was also tested in a sensitivity analysis.

## 3. Results

As shown in Table 1 and Table S2, no differences were noted in the distribution of sex, age, steroid use, area of residence, occupation, or disease history between non-flushers (N = 362) and flushers (N = 445); however, fewer flushers reported drinking habit (Table 1). No significant differences were observed in the number of COVID-19 vaccinations (Table S3). As of May 31, 2023, 147 non-flushers (40.6%) and 159 flushers (35.7%) had experienced COVID-19 (p = 0.16, chi-squared test) (Table S3). As shown in Table S4, the initial infection tended to occur earlier in non-flushers than in flushers (p = 0.057, Spearman’s partial rank correlation coefficient with adjustments for sex and age). The number of self-diagnosed cases tended to be higher in the non-flusher group (10.9%) than in the flusher group (6.9%) (p = 0.058, chi-square test). “Home” was the most common place where infection was suspected (non-flushers, 37.4%; flushers, 36.5%), whereas “Restaurant” was not common in either group (12.9% and 11.9%, respectively). Nine participants (2.5% of the total and 6.1% of those with COVID-19 experience) were non-flushers, and two (0.4% and 1.3%, respectively) were flushers hospitalized due to COVID-19 (p = 0.015 and 0.030, respectively, Fisher’s exact test).

**Table 1:** Characteristics of the participants.

	**Non-flusher ** **(362)**		**Flusher ** **(445)**		P*

	**N**	**%**	**N**	**%**	
**Sex**					*0.2946*
Male	190	52.5%	250	56.2%	
Female	172	47.5%	195	43.8%	
**Age (years)**					*0.463*
20–39	93	25.7%	112	25.2%	
40–59	195	53.9%	226	50.8%	
≥60	74	20.4%	107	24.0%	
**Steroid use**					*0.6695*
No	323	89.2%	388	87.2%	
Yes	21	5.8%	30	6.7%	
Not sure	18	5.0%	27	6.1%	
Prefecture of residence**					*0.185*
Non-endemic	175	48.3%	236	53.0%	
Endemic	187	51.7%	209	47.0%	
**Occupation**					*0.158*
Government officials	29	8.0%	28	6.3%	
Students, faculty, and staff	22	6.1%	42	9.4%	
Housework	23	6.4%	22	4.9%	
Interpersonal service	39	10.8%	59	13.3%	
Local government	41	11.3%	65	14.6%	
Medical and nursing care	40	11.0%	33	7.4%	
Office work	82	22.7%	102	22.9%	
Others	86	23.8%	94	21.1%	
**Habitual drinking**					*<0.001*
No (≤1/week)	126	34.8%	286	64.3%	
Yes (>1/week)	236	65.2%	159	35.7%	

*Chi-square test. **Hokkaido, Tokyo, Kanagawa, Aichi, Osaka, Kyoto, and Fukuoka were defined as endemic areas of residence.

**Fig. 1:**
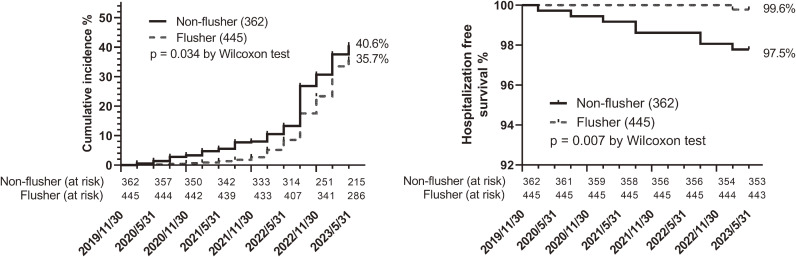
Cumulative incidence rate of COVID-19 and related hospitalization-free survival. Data were obtained from a web-based retrospective survey conducted from August 7 to 27, 2023. The number of participants is shown in parentheses.

**Fig. 2:**
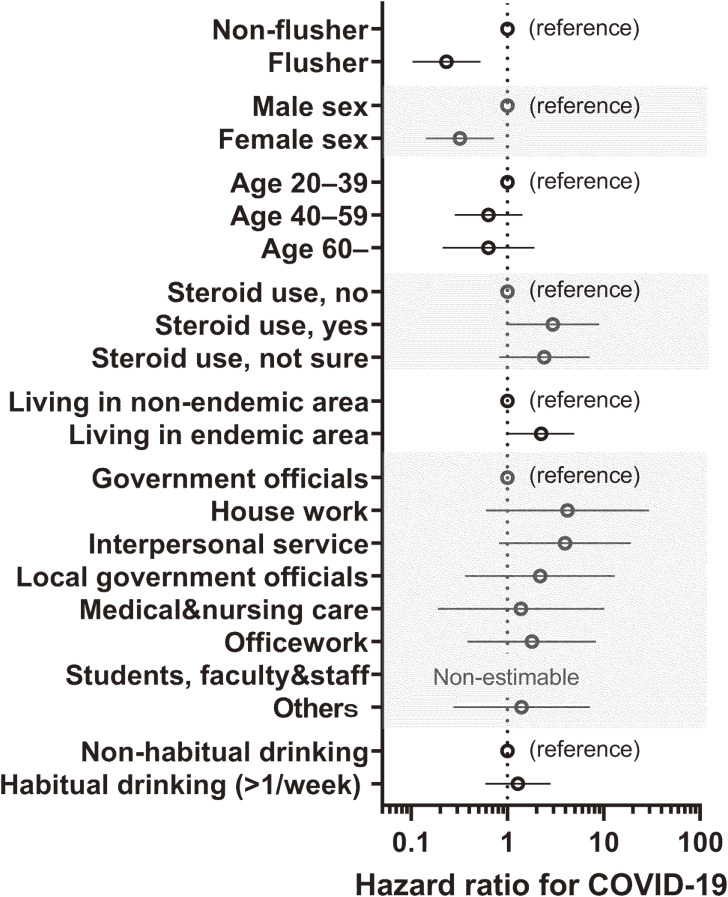
Hazard ratio for COVID-19 incidence during the first 21-month observation period beginning in December 2019. The hazard ratios estimated using Model 2 in Table 2 were visualized.

As shown in Fig. 1, a survival analysis covering a 42-month period from December 2019 to May 2023 showed fewer morbidities and hospitalization events in flushers (p = 0.03 and 0.01, respectively, generalized Wilcoxon test). In the COX proportional hazards model, which covered a 21-month observation period from December 2019 to August 2021, the flushing characteristics were associated with a lower risk of COVID-19 (Table 2) in all statistical models. Model 1, which included sex, age categories, steroid use, and area of residence as covariates, showed the best fit (AIC = 463), with an estimated hazard ratio (HR) of 0.21 and a 95% confidence interval (CI) of 0.095–0.461. Model 2, which included additional covariates such as occupation and habitual drinking, showed a similar relationship. The forest plot indicating the protective effect of female sex and the risk of living in the endemic area estimated by Model 2 is shown in Fig. 2.

**Table 2:** Effect of flushing on COVID-19 incidence during the first 21-month period beginning in December 2019.

	**Crude**		**Model 1**		**Model 2**	
	**AIC = 466**		**AIC = 463**		**AIC = 467**	
	**β**	***p***	**β**	***p***	**β**	***p***
Flusher (vs. non-flusher)	**−1.49**	***0.0002***	**−1.57**	***0.0001***	**−1.46**	***0.0004***
Female sex (vs. male sex)			−0.98	*0.0094*	−1.14	*0.0062*
Age (years)						
20–39 (reference)						
40–59			−0.28	*0.4631*	−0.45	*0.2728*
≥60			−0.32	*0.5067*	−0.46	*0.4148*
Steroid use						
No (reference)						
Yes			1.00	*0.0671*	1.08	*0.0572*
Not sure			0.88	*0.1009*	0.88	*0.1106*
Prefecture of residence						
Endemic (vs. non-endemic)			0.69	*0.0544*	0.81	*0.0446*
Occupation						
Government officials (reference)						
Students, faculty, and administrative staff					−13.47	*0.9848*
Housework					1.43	*0.1500*
Interpersonal service					1.38	*0.0871*
Local government officials					0.78	*0.3944*
Medical and nursing care					0.32	*0.7502*
Office work					0.58	*0.4575*
Others					0.34	*0.6859*
Habitual drinking (>1/week)					0.25	*0.5320*

The partial regression coefficient, β, was estimated with Cox proportional hazard models.

When self-diagnosed cases were considered unaffected, the association weakened in the survival analysis for the entire 42-month period (Fig. S4; p = 0.08), and a stronger association was estimated using the Cox proportional hazards model (adjusted HR = 0.20, p = 0.0002 in Model 1). Other sensitivity analyses yielded similar result as shown in Table 2: HR (95% CI) of 0.209 (0.095–0.461) (AIC = 465) for Model 1 additionally adjusted for BMI, 0.206 (0.093–0.457) (AIC = 472) for disease history (diabetes, respiratory disease, heart disease, allergic disease, collagen disease, and hepatic disease), and 0.211 (0.095–0.468) (AIC = 468) for smoking habit. As depicted in Fig. S5, the interaction between habitual drinking and flushing was not significant; however, the estimates suggested that drinking habits were more hazardous among non-flushers (p = 0.25).

## 4. Discussion

To test our hypothesis that the *ALDH2* rs671 variant is protective against COVID-19, we conducted a web-based retrospective survey on the indicator characteristics of the variant (skin flushing after drinking) and timing of COVID-19 infection. We found that flushing was associated with a lower risk of morbidity and hospitalization due to COVID-19. Attempts at multivariate-adjusted estimation revealed an HR of 0.21 for participants who were aware of flushing during the first 21-month observation period ending on August 31, 2021, just before the implementation of two-dose vaccination nationwide.

Here, we present evidence supporting the hypothesis that rs671 has evolutionarily expanded owing to its infection defense property. This property has been confirmed in *Aldh2*-knockout mice with *Mycobacterium tuberculosis* and *Francisella tularensis* infection challenges at the physiological level [18]. Berry et al. (2023) suggested that, following bacterial invasion, more accumulation of endogenously produced aldehydes was observed in *Aldh2*-knockout mice than in wild-type mice, resulting in a higher tolerance to bacterial infection. Endogenous aldehydes, such as formaldehyde, 4-HNE, and acrolein, have been reported to stimulate innate immunity, including the macrophage response and IgM production [16, 18, 31]. Furthermore, the direct bacteriostatic effects of such aldehydes have been documented [18] and well-established in the field of botany; green leaf volatiles are endogenous aldehydes in plants, which induce resistance against pathogens [32]. In animals, endogenous formaldehyde reportedly exists in a concentration range sufficient to inhibit bacterial growth (>25 µM) [18], even under normal conditions [15], and thus, higher formaldehyde concentrations in variant allele carriers of rs671 may confer protective effects against SARS-CoV-2 infection. Moreover, endogenous 4-HNE may enhance autophagy [33], which is important for the regulation of SARS-CoV-2 infection [34], suggesting the possibility of additional mechanisms. These mechanisms may explain the theory that reduced aldehyde metabolism in rs671 carriers results in viral resistance due to increased concentrations of endogenous aldehydes.

In this study, flushing was found to be protective against COVID-19-related hospitalization. This finding can be partially explained by the same mechanism associated with low morbidity, because the infection-defensive property results in less viral entry. In addition, findings on humoral immunity suggest the possibility of another mechanism. Severe COVID-19 infection has been reported to be associated with a larger expansion of antibody-secreting cells and early production of high concentrations of SARS-CoV-2-specific neutralizing antibodies [35, 36]. We found that the concentrations of specific IgG antibodies after COVID-19 vaccination were low in rs671 variant carriers (the IgG levels 2 weeks after administration of the second dose were estimated to be 3090, 1843, and 1098 BAU/mL for participants carrying wild-homozygous, heterozygous, and variant-homozygous genes, respectively) [37]. These findings suggest that rs671 may be associated with a lower acquired humoral immunity response, that is, the suppression of an excessive immune response, resulting in fewer hospitalization events.

A genome-wide association study (GWAS) suggested that a unique variant allele in East Asians, the rs60200309 A allele, which is associated with decreased expression of *DOCK2*, is a risk allele for COVID-19 in a Japanese cohort of 2393 patients and 3289 unaffected controls recruited at 100 affiliated hospitals between April 2020 and January 2021. A relatively small but robust odds ratio (95% CI) of 1.25 (1.09–1.41) was observed, exceeding the significance threshold (p < 5.0 × 10^−8^), whereas rs671 was not detected [38]. Based on the result of the current study, the effect of rs671 is expected to be greater but not robust enough to be found in GWAS, where adjustment for covariates is limited. Based on previous study findings, rs671 is expected to influence several COVID-19-related traits, such as food preference and underlying metabolic conditions and diseases [39–41], resulting in the inclusion of various noises.

The study assumes that flushing predicts rs671 based on previous studies showing its high accuracy, 88–94% [28–30], and an overwhelming association between flushing and rs671 among other gene polymorphisms [42]. Based on the distribution of rs671 and the frequency of flushers in previous studies in Japan, the rate of flushers was expected to be 46%–51% [39, 43, 44]; however, the proportion in the current study was as high as 55%. We speculate that this was because of false positives, especially in elderly individuals, for the following reason. As it is empirically known that non-flushers begin to experience mild skin flushing at an older age, we used a questionnaire about flushing around the time of the first drink as a surrogate marker for rs671. However, a certain percentage of participants may have skipped over the notes of “around the time of the first drink” and responded. This misclassification may have resulted in a lower estimate of the HR. Hsiao et al. (2019) found that 23.5% of non-carriers of the rs671 variant allele over 60 years of age and 17.4% of those under 60 years of age reported flushing (percentage of false positives), whereas flushing in our study was the most common among participants aged 60 years (59%) [45], indicating that misclassification in elderly individuals is a general limitation for estimating rs671 genotypes using the flushing phenomenon. In addition, the participants in this survey may have been biased toward those who proficiently use social networking sites, and as the survey recruitment was spread through friends of the contributor, who is a government employee and a resident of Tokyo, the socioeconomic status and other characteristics of the participants may have been biased, and the generalizability of the results is limited, although it is unlikely that the association was distorted. Similarly, the high infection rate in this study (39 cumulative events as of 8/31/2021 in the current study vs. 9.4 expected events calculated from national statistics; Table S5) indicates a selection bias toward those with COVID-19 experience. However, it is unlikely that the association has altered.

## 5. Conclusions

To determine whether the *ALDH2* rs671 variant is protective against COVID-19, we retrospectively investigated the association between the flushing trait, a surrogate marker for rs671, and the timing of COVID-19 development using a web-based survey tool. We found that flushers had a lower risk of COVID-19 morbidity and hospitalization than non-flushers, indicating that the rs671 variant is a possible protective factor against COVID-19. Our study provides useful information for controlling future infection, understanding diversity, and developing personalized healthcare.

## Abbreviations

4-HNE: 4-hydroxy 2-nonenal
*ALDH2*: aldehyde dehydrogenase 2
BMI: body mass index
COVID-19: coronavirus disease 2019
CI: confidence interval
HR: hazard ratio
GWAS: genome-wide association study

## Supplementary information

**Additional file 1:** **Figure S1.** Cumulative number of COVID-19 cases or deaths per 100,000 individuals. **Table S1.** Questionnaire distributed to the respondents. **Figure S2.** Plot of log (−log (COVID-19-free survival ratio)) versus log (days of COVID-19-free survival). **Figure S3.** Number of COVID-19 vaccinations in the Japanese population. **Table S2.** Disease history of participants. **Table S3.** Information on COVID-19 vaccination and events of the participants. **Table S4.** Number of patients who have COVID-19 for the first time during the 42-month observation period. **Figure S4.** Cumulative incidence rate of COVID-19 with self-diagnosed cases considered unaffected. **Figure S5.** Cumulative incidence rate of COVID-19 stratified by habitual drinking. **Table S5.** Expected number of COVID-19 cases as of 2021/8/31 by age distribution.

## Declarations

### Availability of data and materials

The datasets generated and/or analyzed in the current study are not publicly available to protect the privacy of the participants, but they are available from the corresponding author on reasonable request.
