# Supplementary material for: Asian flush is a potential protective factor against COVID-19: a web-based retrospective survey in Japan
Source: Environ Health Prev Med. 2024 Mar 9;29:14. doi: 10.1265/ehpm.23-00361 (PMC10937249; doi:10.1265/ehpm.23-00361)
Supplement: Supplementary file 1 — Additional file 1: Figure S1. Cumulative number of COVID-19 cases or deaths per 100,000 individuals. Table S1. Questionnaire distributed to the respondents. Figure S2. Plot of log (−log (COVID-19-free survival ratio)) versus log (days of COVID-19-free survival). Figure S3. Number of COVID-19 vaccinations in the Japanese population. Table S2. Disease history of participants. Table S3. Information on COVID-19 vaccination and events of the participants. Table S4. Number of patients who have COVID-19 for the first time during the 42-month observation period. Figure S4. Cumulative incidence rate of COVID-19 with self-diagnosed cases considered unaffected. Figure S5. Cumulative incidence rate of COVID-19 stratified by habitual drinking. Table S5. Expected number of COVID-19 cases as of 2021/8/31 by age distribution. [file ehpm-29-014-s001.docx]

**Figure S1. Cumulative number of COVID-19 cases or deaths per 100,000 individuals.**

Data were obtained from the World Health Organization database (<https://covid19.who.int/WHO-COVID-19-global-data.csv>). The numbers represent occurrences per population of cases (left panels) or deaths (right panels) through October 2023 (A) and September 2021 (B and C at different scales), respectively.

| Table S1. Questionnaire distributed to the respondents. | | |
| --- | --- | --- |
| Questions | | Answers |
| Are you the person in question? | | It is the person in question.  (Only option to proceed) |
| Biological sex | | 1. Male 2. Female 3. Other |
| Prefecture of residence | | Select from 47 prefectures |
| Occupation | | 1. Jobs that involve serving customers for relatively long periods of time, such as waitstaff in restaurants and other interpersonal services, and outside salespeople who interact with people. 2. Medical and care workers 3. Office workers excluding government employees, such as indoor sales and distributors. 4. School staff and students 5. Government officials (excluding school staff) 6. Local officials (excluding school staff) 7. Housework, Others |
| Age: As of August 1, 2023 | | 1. 0-19 years old 2. 20-29 years old 3. 30-39 years old 4. 40-49 years old 5. 50-59 years old 6. 60-69 years old 7. 70-79 years old 8. Over 80 years old |
| BMI = body weight (kg) ÷ height (m)^2^  Please enter your BMI from your health checkup or calculate it at the website below.  <https://www.e-healthnet.mhlw.go.jp/information/metabolic/bmi_check.html> | |  |
| Smoking habits:  Answer about your status after 2020. Please select the type of smoking device. | | 1. No smoking 2. Paper cigarettes 3. Heated cigarettes (using tobacco leaves, e.g. iQOS, PloomTech, Glo) 4. Electronic cigarettes without nicotine and tar |
| Drinking habits: Answer about your status after 2020. Please select the frequency of drinking alcoholic beverages. | | 1. Every day 2. At least 4 times a week 3. 2-3 times a week 4. 2-4 times a month 5. Once a month 6. Don’t drink |
| Amount of alcohol consumed (net alcohol):  Please answer the amount of pure alcohol on an average day by referring to the table. You can also use the website for calculation. https://www.suntory.co.jp/arp/alcohol_calculation/  For example, if you do not drink, enter "0".  For 1 regular can (350 mL) of beer (14 g) and 1 regular can (350 mL) of whisky and soda (20 g), enter “34”. |  | |
| Sensitivity to alcohol:  Recall the physical reaction around the time of first drink. | 1. Tolerant to alcohol (no flushing after drinking) 2. Intolerant to alcohol (flushing after drinking) 3. I can't drink (flushing after a little drinking) | |
| Medical History  (multiple choice) | 1. Diabetes mellitus 2. Cardiac disease 3. Respiratory disease 4. Allergic disease 5. Collagen disease 6. Hepatitis (including hepatitis A to E virus infections) 7. Fatty liver 8. Gallstones, gallbladder dysfunction, and cholecystectomy 9. None | |
| Steroid medication status: Answer about the situation after 2020. | 1. Taking steroids 2. Not taking steroids 3. I am not sure | |
| Number of COVID-19 vaccinations: If you have forgotten, check your vaccination app or vaccination certificate. | 1. Unvaccinated 2. 1 dose 3. 2 doses 4. 3 doses 5. 4 doses 6. 5 doses 7. 6 doses 8. I cannot remember | |
| COVID-19 history | 1. None 2. Once 3. Twice 4. More than twice | |
| The rationale for a diagnosis of the first COVID-19 | 1. Antigen test 2. PCR test 3. Physician’s diagnosis 4. Self-diagnosis based on symptoms 5. I cannot remember | |
| Time of the first COVID-19: If the disease period is spread over two categories, please select the one with the longer duration. | 1. January-February 2020 2. March - May 2020 3. June-August 2020 4. September - November 2020 5. December 2020 - February 2021 6. March - May 2021 7. June-August 2021 8. September - November 2021 9. December 2021 - February 2022 10. March - May 2022 11. June-August 2022 12. September - November 2022 13. December 2022- February 2023 14. March - May 2023 15. June - August 2023 16. I don't remember | |
| Hospitalization experience due to the first COVID-19: | 1. No 2. Yes | |
| The location where the first infection may have occurred. | 1. Restaurant 2. Home 3. Workplace/school 4. Other | |
| The rationale for a diagnosis of the second COVID-19 | 1. Antigen test 2. PCR test 3. Physician’s diagnosis 4. Self-diagnosis based on symptoms 5. I cannot remember | |
| Time of the second COVID-19: If the disease period is spread over two categories, please select the one with the longer duration. | 1. January-February 2020 2. March - May 2020 3. June-August 2020 4. September - November 2020 5. December 2020 - February 2021 6. March - May 2021 7. June-August 2021 8. September - November 2021 9. December 2021 - February 2022 10. March - May 2022 11. June-August 2022 12. September - November 2022 13. December 2022- February 2023 14. March - May 2023 15. June - August 2023 16. I don't remember | |
| Hospitalization experience due to the second COVID-19: | 1. No 2. Yes | |
| The location where the second infection may have occurred. | 1. Restaurant 2. Home 3. Workplace/school 4. Other | |
| The rationale for a diagnosis of the third COVID-19 | 1. Antigen test 2. PCR test 3. Physician’s diagnosis 4. Self-diagnosis based on symptoms 5. I cannot remember | |
| Time of the third COVID-19: If the disease period is spread over two categories, please select the one with the longer duration. | 1. January-February 2020 2. March - May 2020 3. June-August 2020 4. September - November 2020 5. December 2020 - February 2021 6. March - May 2021 7. June-August 2021 8. September - November 2021 9. December 2022- February 2023 10. March - May 2022 11. June - August 2023 12. September - November 2022 13. December 2022- February 2023 14. March - May 2023 15. June - August 2023 16. I don't remember | |
| Hospitalization experience due to the third COVID-19: | 1. No 2. Yes | |
| The location where the third infection may have occurred. | 1. Restaurant 2. Home 3. Workplace/school 4. Other | |


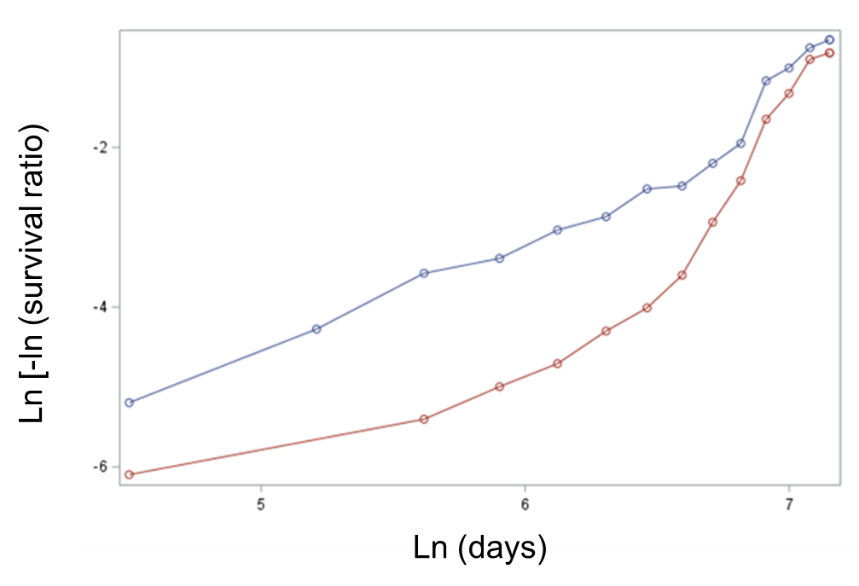


**Figure S2. Plot of log (-log (COVID-19-free survival ratio)) versus log (days of COVID-19-free survival).**
Hazard proportionality was assessed using the parallelism between the non-flusher (blue line) and flusher (red line) curves to determine the suitability of the Cox proportional hazard model.

**Figure S3. Number of COVID-19 vaccinations in the Japanese population.**

A public database created by the Japanese Ministry of Health, Labour, and Welfare was visualized. The dotted line indicates 2021/8/31, which is defined as the boundary between the first and second halves of the observation period.

| Table S2. Disease history of participants | | | | | |
| --- | --- | --- | --- | --- | --- |
|  | Non-flusher (362) | | Flusher (445) | |  |
|  | N | % | N | % | *P-value** |
| Diabetes mellitus | 9 | 2.5% | 22 | 4.9% | *0.096* |
| Respiratory disease | 29 | 8.0% | 37 | 8.3% | *0.898* |
| Cardiac disease | 8 | 2.2% | 12 | 2.7% | *0.821* |
| Allergic disease | 57 | 15.7% | 77 | 17.3% | *0.570* |
| Collagen disease | 0 | 0.0% | 3 | 0.7% | *0.257* |
| Hepatitis | 7 | 1.9% | 9 | 2.0% | *1.000* |
| Fatty liver | 42 | 11.6% | 61 | 13.7% | *0.397* |
| Gallstones, gallbladder dysfunction, cholecystectomy | 13 | 3.6% | 13 | 2.9% | *0.690* |
| *Fisher's exact test. |  |  |  |  |  |

| Table S3. Information on COVID-19 vaccination and events of the participants. | | | | | |
| --- | --- | --- | --- | --- | --- |
|  | Non-flusher | | Flusher | |  |
|  | N | （%） | N | （%） | *P-value*** |
| Number of COVID-19 vaccination |  |  |  |  |  |
| 0 | 23 | 6.4% | 31 | 7.0% | 0.727 |
| 1 | 3 | 0.8% | 2 | 0.4% |  |
| 2 | 40 | 11.0% | 57 | 12.8% |  |
| 3 | 126 | 34.8% | 155 | 34.8% |  |
| 4 | 90 | 24.9% | 106 | 23.8% |  |
| 5 | 54 | 14.9% | 59 | 13.3% |  |
| 6 | 24 | 6.6% | 35 | 7.9% |  |
| Forgotten | 2 | 0.6% | 0 | 0.0% |  |
| COVID-19 experience counts |  |  |  |  |  |
| 0 | 215 | 59.4% | 286 | 64.3% | 0.375 |
| 1 | 135 | 37.3% | 147 | 33.0% |  |
| 2 | 11 | 3.0% | 12 | 2.7% |  |
| >2 | 1 | 0.3% | 0 | 0.0% |  |
| COVID-19 Diagnostic Rationale* |  |  |  |  |  |
| PCR/antigen test/physician's diagnosis | 130 | 88.4% | 148 | 93.1% | 0.511 |
| Self-diagnosis | 16 | 10.9% | 11 | 6.9% |  |
| Forgotten | 1 | 0.7% | 0 | 0.0% |  |
| Suspected location for infection* |  |  |  |  |  |
| Restaurant | 19 | 12.9% | 19 | 11.9% | 0.974 |
| Home | 55 | 37.4% | 58 | 36.5% |  |
| Workplace/school | 31 | 21.1% | 37 | 23.3% |  |
| Other | 42 | 28.6% | 45 | 28.3% |  |
| Hospitalization* |  |  |  |  |  |
| No | 138 | 93.9% | 157 | 98.7% | 0.0153 |
| Yes*** | 9 | 6.1% | 2 | 1.3% |  |
| *Among 147 non-flushers or 159 flushers. **Fisher's exact test. ***10 hospitalizations out of 11 occurred at the first infection, except one non-flusher at the second infection. | | | | | |

| Table S4. Number of patients who have COVID-19 for the first time during the 42-month observation period. | | | | | | |
| --- | --- | --- | --- | --- | --- | --- |
| Checkpoints |  | Non-flusher (N = 362) | |  | Flusher (N = 445) | |
|  |  | COVID-19 | Hospitalization |  | COVID-19 | Hospitalization |
| 2019/12/1 |  | 0 (0) | 0 |  | 0 (0) | 0 |
| 2020/2/28 |  | 2 (1) | 1 |  | 1 (1) | 0 |
| 2020/5/31 |  | 3 (1) | 0 |  | 0 (0) | 0 |
| 2020/8/31 |  | 5 (0) | 1 |  | 1 (0) | 0 |
| 2020/11/30 |  | 2 (0) | 1 |  | 1 (0) | 0 |
| 2021/2/28 |  | 5 (0) | 1 |  | 1 (0) | 0 |
| 2021/5/31 |  | 3 (0) | 0 |  | 2 (0) | 0 |
| 2021/8/31 |  | 8 (1) | 2 |  | 2 (0) | 0 |
| 2021/11/30 |  | 1 (0) | 0 |  | 4 (2) | 0 |
| 2022/2/28 |  | 9 (0) | 0 |  | 11 (0) | 0 |
| 2022/5/31 |  | 10 (4) | 0 |  | 15 (2) | 0 |
| 2022/8/31 |  | 49 (3) | 2 |  | 40 (0) | 0 |
| 2022/11/30 |  | 14 (1) | 0 |  | 26 (2) | 1 |
| 2023/2/28 |  | 25 (3) | 1 |  | 45 (2) | 1 |
| 2023/5/31 |  | 11 (2) | 0 |  | 10 (2) | 0 |
| Total event |  | 147 | 9 |  | 159 | 2 |

Numbers in parentheses indicate the number of cases with self-diagnosis.

**Figure S4. Cumulative incidence rate of COVID-19 with self-diagnosed cases considered unaffected.**

The numbers of observations are shown in parentheses.

**Figure S5. Cumulative incidence rate of COVID-19 stratified by habitual drinking.**

Habitual drinking was divided into two (A) and three categories (B).

| Table S5. Expected number of COVID-19 cases as of 2021/8/31 by age distribution. | | | | | | | |
| --- | --- | --- | --- | --- | --- | --- | --- |
|  | Current study | |  |  |  | National statistics* (×1000) | |
|  |  | Cumulative number of COVID-19 cases | | | |  |  |
|  | Age distribution | First | Second | Total | Expected | Age distribution | Cumulative COVID-19 |
| 20–29 | 89 | 4 | 1 | 5 | 2.3 | 12640 | 331 |
| 30–39 | 116 | 7 | 1 | 8 | 1.8 | 13938 | 215 |
| 40–49 | 151 | 7 |  | 7 | 1.7 | 17941 | 205 |
| 50–59 | 270 | 11 |  | 11 | 2.6 | 17035 | 165 |
| 60–69 | 147 | 7 | 1 | 8 | 0.8 | 15278 | 82 |
| 70–79 | 19 | 0 |  | 0 | 0.1 | 16390 | 62 |
| 80–89 | 15 | 0 |  | 0 | 0.1 | 9405 | 42 |
| Total | 807 | 36 | 3 | 39 | 9.4 | 102627 | 1101 |
| *Public data from the Japanese Ministry of Health, Labour, and Welfare and the Ministry of Internal Affairs and Communications as of 2021/8/31 | | | | | | | |
